# Universal access: user needs for immersive captioning

**DOI:** 10.1007/s10209-021-00828-w

**Published:** 2021-07-15

**Authors:** Chris. J. Hughes

**Affiliations:** School of Computer Science, University of Salford, Manchester, UK

**Keywords:** Accessibility, User-centric requirements, Testing, VR, Immersive video, Captions

## Abstract

This article focuses on building a prototyping for immersive captioning following a user-centric approach. This methodology is characterised by following a bottom-up approach, where usability and user needs are at the heart of the development. Recent research on user requirements for captioning in immersive environments has shown that there is both a need for improvement and a wealth of research opportunities. The final aim is to identify how to display captions for an optimal viewing experience. This work began four years ago with some partial findings. We build from the lessons learnt, focussing on the user-centric design requirements cornerstone: prototyping. Our prototype framework integrates methods used in existing solutions aiming at instant contrast-and-compare functionalities. The first part of the article presents the state of the art for user requirements identifying the reasons behind the development of the prototyping framework. The second part of the article describes the two-stage framework development. The initial framework concept answered to the challenges resulting from the previous research. As soon as the first framework was developed, it became obvious that a second improved solution was required, almost as a showcase on how ideas can quickly be implemented for user testing, and for users to elicit requirements and creative solutions. The article finishes with a list of functionalities, resulting in new caption modes, and the opportunity of becoming a comprehensive immersive captions testbed, where tools such as eye-tracking, or physiological testing devices could be testing captions across any device with a web browser.

## Introduction

Immersive media technologies, like virtual reality (VR) and 360º video, have rapidly grown in popularity due to the availability of consumer-level head-mounted displays (HMDs). This influences not only the entertainment sector, but also other key sectors of society, like education, arts and culture [1] especially during the times of the COVID-19 pandemic [2] when people are less able to travel. In this context, 360º videos have become a simple and cheap, yet effective and hyper-realistic, medium to provide VR experiences. Due to this potential, the scientific community and industry have devoted significant resources to developing new solutions in terms of many relevant aspects, like authoring and playback hardware and media players. This has led to increased demand for the production and consumption of 360º videos, and major platforms, like YouTube, Facebook and news platforms such as The New York Times, currently provide 360º videos in their service offerings [1].

It is a logical development that these new media environments are accessible for all to fulfil existing accessibility legislation in most world regions. This follows the requirements from signing the UN Human Rights CRPD (Convention of the Rights of People with Disabilities) with the motto ‘*nothing about us without us’*. This user-centric approach is at the heart of Human Rights towards minorities full democratic participation in society—which in the twenty-first century depends on access to media [3]. It is within this user-centric approach that requirements were gathered to develop captions in immersive environments. Even though development of media access services and workflows started almost in parallel to the development of media content, there is always a pull from mainstream media production before accessibility services such as captioning catch up. With the development of different VR content genres, and personal preferences, different captions need have arisen. Using a prototyping framework that allows for fast visualisation and testing is the way forward to design new caption designs.

## Background

New developments in VR technology have led to the development of 360º video players for different platforms such as desktop computers, smartphones and head-mounted displays (HMDs) [4, 5]. As for every service, 360º media consumption experiences need to be accessible. Typically, accessibility has been considered in the media sector as an afterthought, and generally only for mainstream services.

Within traditional media (such as television and movies), there are clear regulations as to how accessibility must be delivered [6]. However, it seems that accessibility for immersive media services is still in its infancy although some projects, such as the EU-funded Immersive Accessibility (ImAc) project [7], have begun to address the need for general accessibility in immersive environments. Their solutions have mainly been to adapt existing accessibility methods, rather than identifying the potential that the new visual and sound environments can offer. In the case of captioning (often referred to as subtitling) early user trials have shown that the users want what they are used to, rather than what they could have. This means rendering the traditional caption (2 lines, ~30 characters wide) into the users view.

More specifically initial user requirements for captions in immersive environments were documented as part of the ImAc Project [8]. According to feedback from focus groups, 360º videos should:

1. Be located in a fixed position and always visible in relation to the users field of view (FoV) and preferably at the bottom;
2. Have a solid background to avoid contrast issues with an unpredictable background;
3. Include a guide which indicates the direction to the speaker when they are outside of the users view. (These could include arrows, a compass or text between brackets).

The study also identified that home users would be willing to accept new approaches for captioning immersive content, such as icons for non-speech information as there are new possibilities and dimensions brought by the new technology. Users also expressed a strong desire for further customisation options. Given the IT possibilities for further caption improvement research has continued departing from the design of a prototyping solution.

This paper discusses a software framework, designed to allow rapid prototyping of different captioning methods, in order to allow new ideas to be tested quickly and easily across different platforms (including desktop, mobile and HMD’s). The tool allows for methods used in existing solutions to be easily contrasted and compared, as well as new ideas quickly implemented for user testing.

Currently, there exist no standard guidelines or implementation for captions in immersive videos, and although many immersive video players now offer the ability to play 360° media, the support for any accessible services is extremely limited. At best the players generally support the implementation of traditional captions fixed within the user’s view [9].

The British Broadcasting Corporation (BBC) was one of the first research organisations to perform user testing with immersive captions [10]. All of their work was based upon projecting traditional captions into the immersive environment, and they evaluated how successful this could be done in scenarios where the captions were:

1. *Evenly Spaced*: Captions repeated at 120° intervals;
2. *Head-locked*: Captions fixed within the users view;
3. *Head-locked with lag*: Captions follow users view, but only for larger head movements;
4. *Appear in front and then fixed*: Captions are placed in the position that the user is looking and remain there until they are removed.

They found that although it was easy to locate the evenly spaced captions, the users much preferred the head-locked options.

A further user study was conducted by the ImAc project, which although identified head-locked captions as a strong preference, it also identified the need to guide users to the source of the caption such as the character speaking. To facilitate this requirement, location information was added to each caption. This allowed for different guiding modes to be developed, such as a directional arrow which could guide the user to where the person speaking was located. However, this did have the drawback that the location was only specified once per caption, and if a person was moving dynamically during this period, the guide could have been wrong [11].

Within VR, captions are now becoming essential in video games. The Last Part of Us: Part II was released in 2020 [12] with a significant focus given to accessibility. Throughout the game the user has the opportunity to enable and customise captions (such as size, font, whether character name is displayed). It also includes a guide arrow to direct the user to the location of the character speaking.

Rothe et al. [13] conducted tests with fixed captions and compared this presentation mode to head-locked captions. Their result did not find that one option was significantly preferred over the other. However, in terms of comfort, fixed captions led to a better result even though fixed captions in general mean that the user may not always be able to see the caption as it may be outside of their view.

A W3C Community group [14] focussed on developing new standards for immersive captioning recently conducted a community survey to gather opinions. A small group of users with different hearing levels (deaf, hard of hearing, and hearing) were asked to evaluate each of the identified approaches for captions within immersive environments.

Head-locked was clearly identified as the preferred choice; however, it was noted that this was most likely as it replicated the experience that users were familiar with. It was also acknowledged that it was difficult for users to properly evaluate new methods theoretically without the opportunity and content to enable them to be experienced properly. Although all agreed that head-locked should be set as default, other choices should be made available. Other suggestions were made which included changing the font size and colour and number of lines (two lines being the default number). Multiple captions should also be in different positions, each being near to the speaker. Therefore, the focus of this research is to produce a framework enabling delivery of the full experience of each captioning mode, in an environment where an extensive user study can be conducted. The framework does not attempt to provide best practice, rather provide an environment where all options can be explored. Therefore, it is possible to create scenarios that are both good and bad.

## Methods

### Implementation

Part of the ambition for a framework which is to be generic enough to enable testing in different environments with a variety of devices is portability. Our implementation is based on web technologies allowing it to be used on any device with a web browser. This includes desktop computers, mobile devices and head-mounted displays (HMDs).

Three.js [15] is a cross-browser JavaScript library and application programming interface (API) used to create graphical processing unit (GPU)-accelerated 3D animations using the JavaScript language on the web without the need for proprietary web browser plugins. In our implementation, it provides high level functionality to WebGL [16] allowing us to define our scene as objects and manipulate them within the space.

**Fig. 1:**
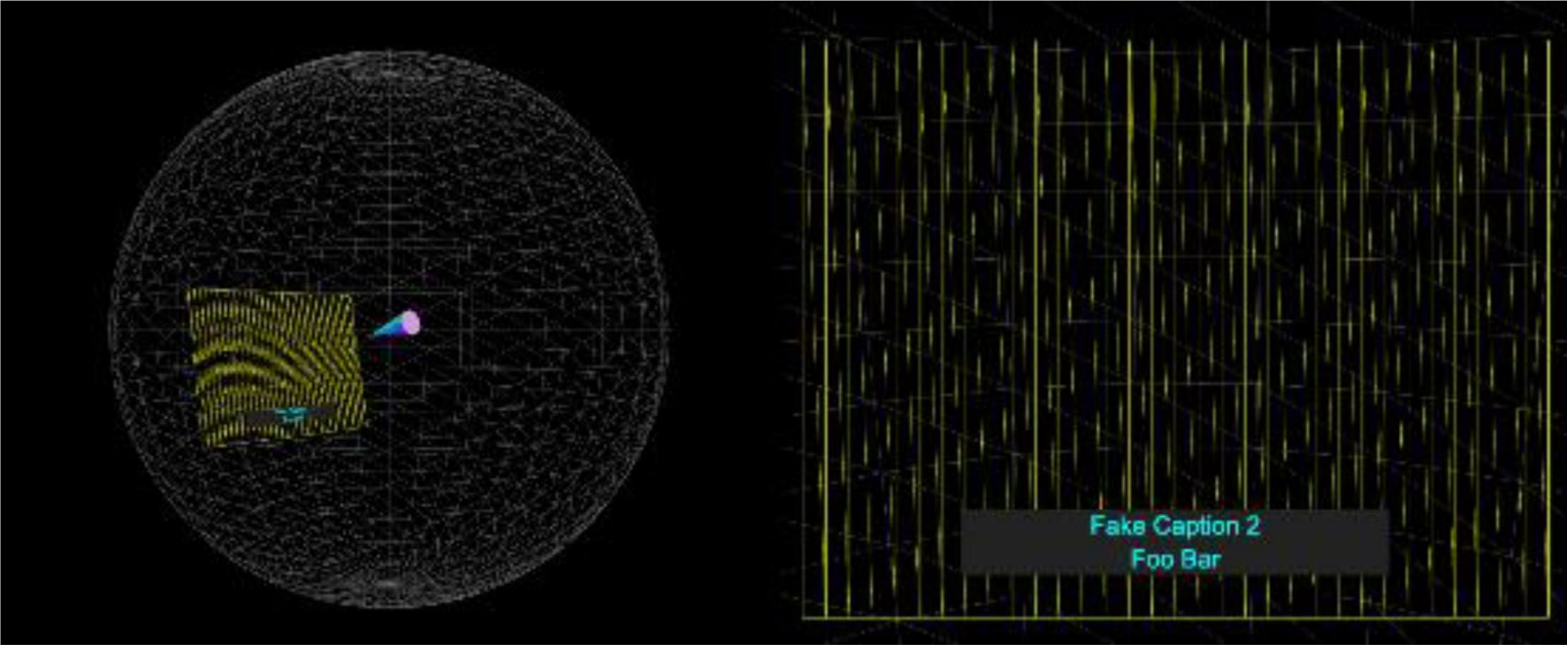
The initial split view of the player allows the user to see both the caption and view window relative to the 360° world and from the user's perspective

In addition, we use a WebVR Polyfill [17], which provides a JavaScript implementation of the WebVR specification [18]. This enables three.js content to work on any platform, regardless of whether or not the browser or device has native WebVR support, or where there are inconsistencies in implementation. The Polyfill's goal is to provide a library so that developers can create content targeting the WebVR API without worrying about what browsers and devices and their users are using. This gives our framework maximum compatibility across a large range of devices. Also, as many devices have limited interfaces, we add an option to automatically play or pause the video when the user enters or leaves VR modes to avoid the need for a play button if controls are available. Our framework allows for the user to switch between several different views or enter VR mode. The default view is a split screen as shown in Fig. 1. This clearly demonstrates how the captions are being rendered and positioned by showing both the user's viewpoint and a representation of the caption and view window within the space.

In order to consume 360° video, the scene contains a *sphere* centred around the origin and it is assumed that the user’s viewpoint remains at the origin. When the framework is not connected to a video, the sphere is rendered as a wireframe; however, once a video has been loaded, the equirectangular video is texture mapper onto the inside of the sphere. As the sphere primitives are generally designed to have a texture mapped to the outside, it is necessary to invert the faces (also known as ‘flipping the normals’) in order to make this work.

Three.js provides a *videoTexture* object, which can connect to any HTML5 video object. Therefore, an HTML5 video is embedded in the webpage, with its display set to ‘none’. The video playback is then managed through JavaScript manipulating the HTML5 video object.

### Architecture design

**Fig. 2:**
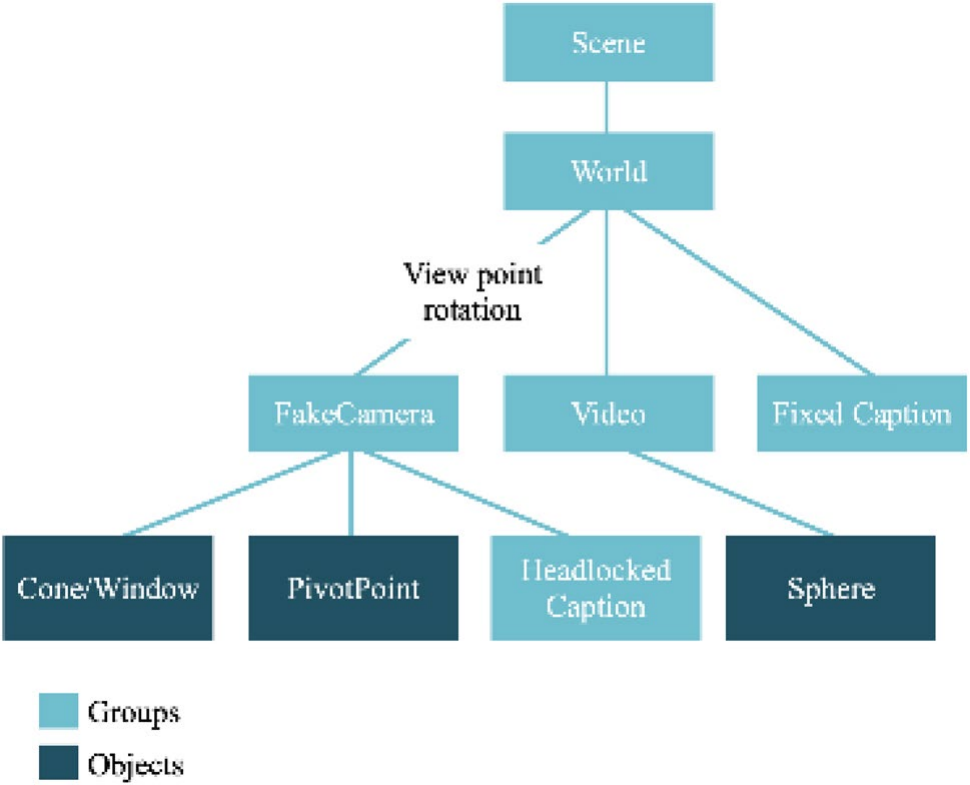
Scene graph of the player framework

The framework was also designed from a bottom-up approach. The departure point is the scene. The basic scene graph of our player is shown in Fig. 2. Inside the main scene container, we first add a world group. This allows to reposition the entire contents of the scene to ensure that the user’s viewpoint is kept at the origin. For example, when using an HMD, the user is automatically given a height which cannot be overridden. Translating the world back to the user’s eye position allows us to keep their view centred. Within the *world,* there are three main components: (1) a video container, (2) a *userView* container and (3) a fixed caption container. The video container is a three.js group which contains the video texture mapped sphere. The *userView* container is a group designed to replicate the behaviour of a camera but which is updated each time the scene is rendered to align with the users’ viewpoint. This allows us to always keep the components within the group locked into the users view, and it contains a caption container, for placing captions which are fixed into the users view window. Finally, within the *world,* there is a fixed caption container which is not updated when the user moves. This allows to place a caption object into either the *userView* group or the fixed-caption group depending on whether the caption is locked in the scene or the users view.

**Fig. 3:**
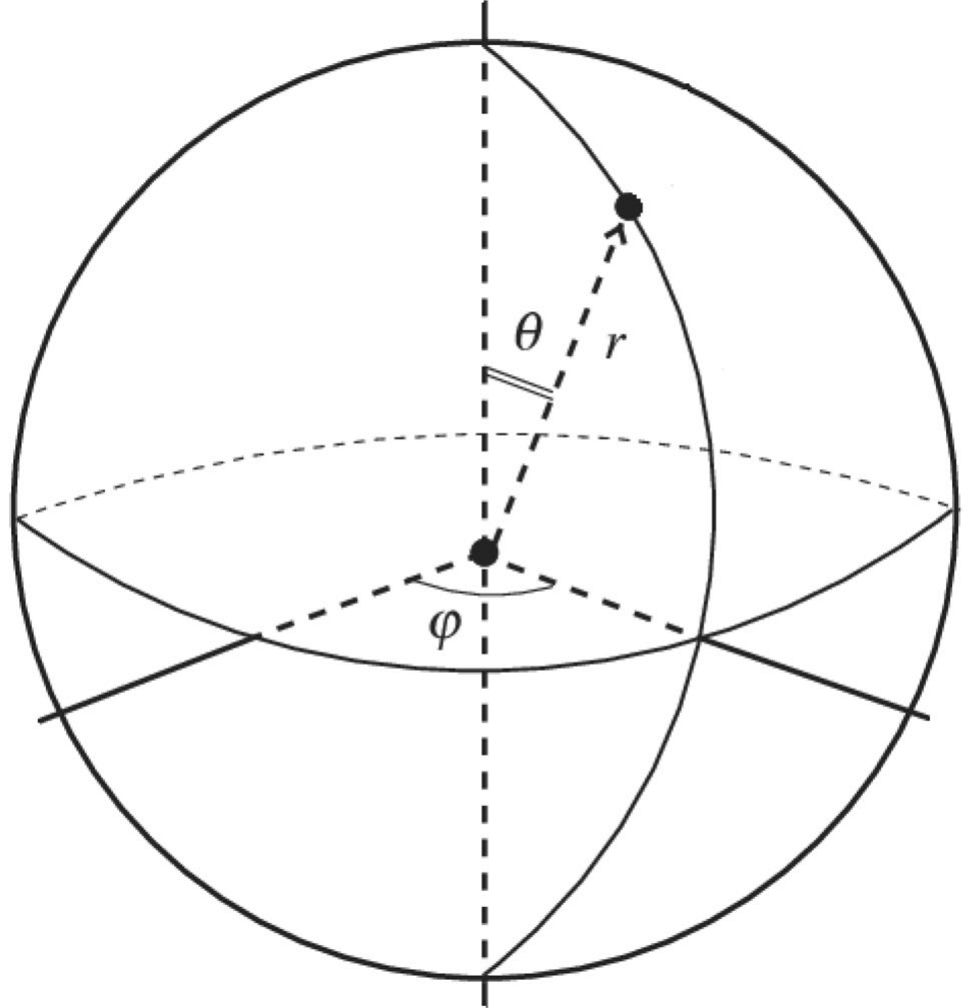
Spherical coordinate system user to position the caption target

A wireframe plane is displayed by default in each view and attached to the *userView* to show the user’s viewpoint and help provide a coordinated understanding of how the views fit together. The *userView* and the fixed-caption container both contain a pivot point ensuring that as they are rotated around the origin, the caption aligns with the video sphere. This allows to simply position the caption anywhere in the video using a spherical coordinate system and by applying a radial distance (r), polar angle (θ) and azimuthal angle (φ) values which are stored in the caption file, as illustrated in Fig. 3.

Two versions of the framework have been implemented. The first provides a robust environment, where existing rendering methods can be contrasted and compared. The second provides an extended environment where new methods and ideas can be evaluated.

### Framework functionalities: contrast and compare

Fundamentally, from our review there are two primary mechanisms for caption rendering: (1) *head-locked* where the caption is rendered relative to the user's viewpoint and (2) *fixed* where the caption is rendered relative to a fixed location in the world, generally at the position of the character speaking.

**Fig. 4:**
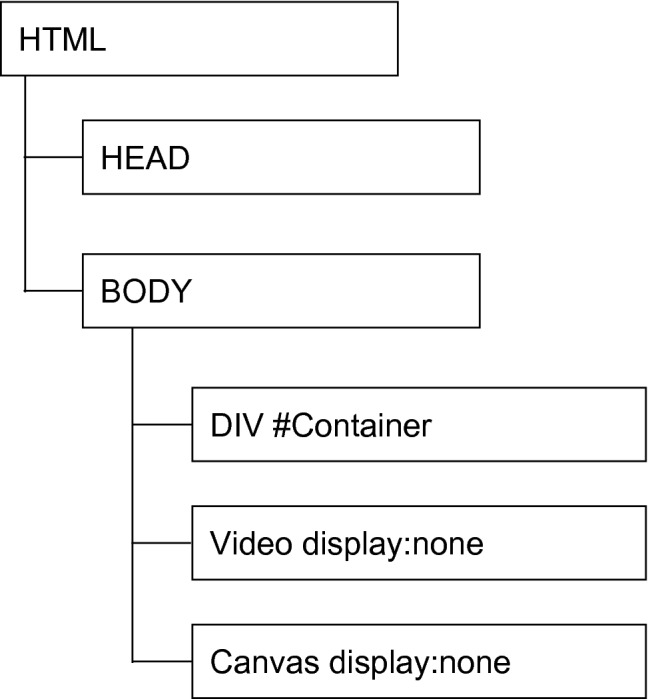
The Document Object Model (DOM) of our player HTML container

Three.js allows for the textures to be generated from any HTML5 canvas object. In addition to the hidden video, our HTML page contains a hidden canvas element which allows us to render any caption using any HTML or CSS styles (as shown in Fig. 4). This canvas texture is then mapped to a *plane* and positioned into the scene.

An update is triggered every time a video frame changes, and the player checks to see if the caption has changed. If there is a new caption, then (1) the canvas is updated to the text and style of the new caption, (2) the texture is updated and, (3) the position of the caption is updated. For a *fixed* caption, this position is attached to its relative position in the scene and placed within the fixed-caption container; however, *head-locked* captions are *userView* object which gets repositioned each time the users’ viewpoint is changed.

Each generated caption is assigned a target location. In the first instance, this is the position that is specified in the caption file. This concept was first used in the ImAc project where a single location was stored for each caption in an extended Timed Text Markup Language (TTML) file [19] and the location is defined in spherical coordinates. Within our player, the user can enable the target position to be displayed in order to help with debugging, and understanding; however, the captions do not necessarily get rendered at this location as the user may have chosen to offset the position, or it may be overridden by the display mode, for example *head-locked* will always render the caption into the users view. On opening, our framework uses a random caption generator to show what is happening in the current display mode. A text string is generated and given a polar position (θ) between −π rad and π rad (−180° to 180°) and azimuthal position (φ) between −0.4 rad and 0.4 rad (~−23° to ~23°) as captions are rarely positioned towards the top or bottom vertical pole.

The user has the opportunity to select from the following default modes:

- *Fixed in Scene, Locked Vertical*: The caption is positioned at the target, but the azimuthal position (φ) is restricted to 0 so that it remains locked to the horizon;
- *Fixed in scene, repeated evenly spaced*: The caption is positioned at the target location and then duplicated at 2π/3 rad (120°) intervals around the horizon;
- *Appear in front, then fixed in scene*: The caption is rendered in the centre of the user’s current view and remains there until the caption is updated;
- *Fixed, position in scene*: The caption is rendered at the target location;
- *Head-locked*: The caption is rendered in the user's view point and is moved in sync with the user to ensure the caption remains statically attached to the view point;
- *Head-locked on horizontal axis only*: The caption is rendered as *head-locked*; however, the azimuthal position (φ) is restricted to 0, ensuring that the caption is always rendered on the horizon;
- *Head-locked on vertical axis only*: The caption is rendered as head-locked; however, the polar position (θ) is locked to the target;
- *Head-locked with lag, animate into view*: The caption is rendered in the *head-locked* position; however, as the users' viewpoint changes, the caption is pulled back towards the *head-locked* position. An animation loop moves the caption incrementally causing it to smoothly animate into view;
- *Head-locked with lag, jump into view*: This is the same as above, except the animation time is reduced to 0, forcing the caption to jump into the users view.

**Fig. 5:**
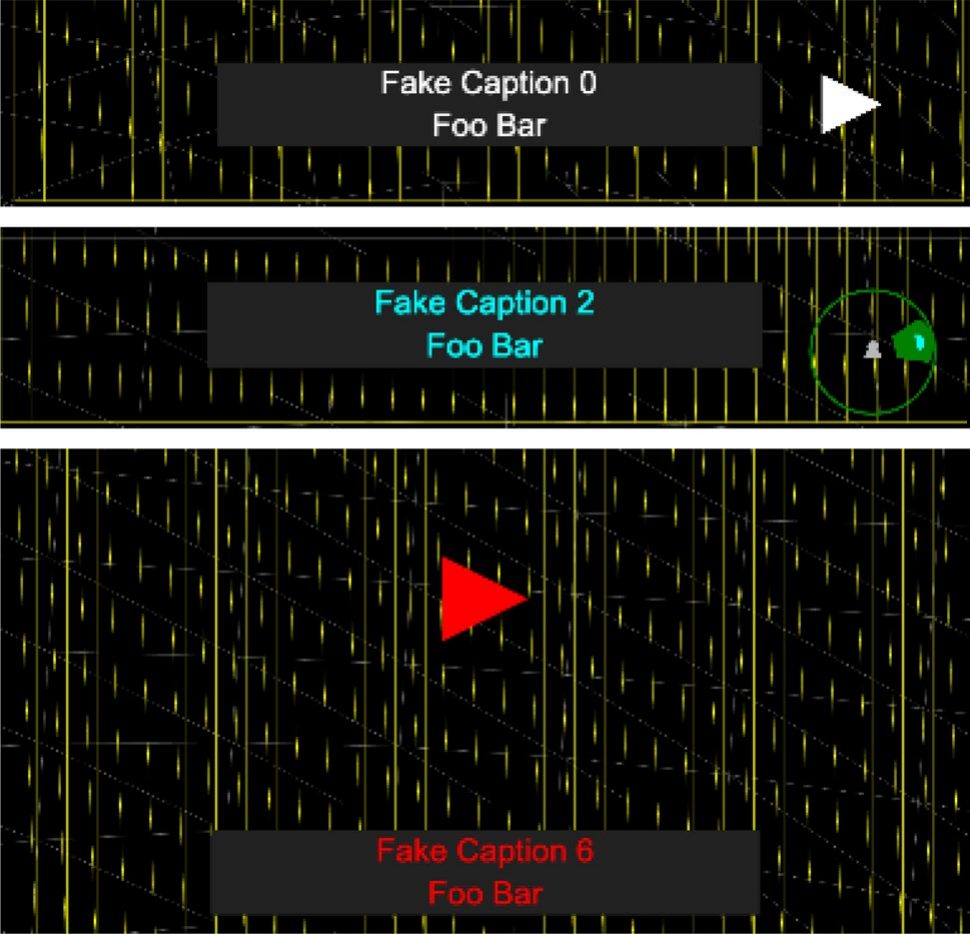
Guide modes (top: ImAc arrow, middle: ImAc radar, bottom: large arrow)

The framework also allows for the comparison of default guiding modes (as shown in Fig. 5). These guide modes always direct the user to the target location as this is the source of the identified action. When the captions are fixed, they therefore direct the user to the caption, whereas when they are *head-locked,* the user can read the caption in their view whilst being directed to the target:

- *ImAc arrow*: An arrow positioned left or right directs the user to the target;
- *ImAc radar*: A radar is shown in the users view. This identifies both the position of the caption and the relative viewing angle of the user;
- *Big arrow*: A large arrow is displayed in the centre of the users view. This guide was developed as part of the framework as a JavaScript demonstrating how new guide modes can be developed.

The JavaScript implementation allows for additional display modes and guide modes to be created quickly by simply creating rules for the caption creation, update and removal.

## New methods

As soon as videos were uploaded to be tested, it became clear that more functionalities were required, which had not been tested, or even requested in the preliminary user tests at ImAc [8].

Due to the large file size of immersive videos, the player was updated to support both HTTP Live Streaming (HLS) [20] using hls.js [21] and Dynamic Adaptive Streaming over HTTP (DASH) [22] streams using dash.js [23]. This massively improves the performance of video playback, where network bandwidth was limited and therefore improves the user experience. Based on anecdotal feedback from the community, additional functionality was added to the player in order to allow further customization.

### Synchronic multiple caption display

Firstly, it was identified that it was necessary to be able to display multiple captions simultaneously. This is because (1) sometimes multiple people are speaking simultaneously and (2) there is a need for captions to remain longer in order to give users time to find and read them.

Our framework was therefore extended to support multiple captions based on a particle system [24] approach. This allows within the framework for the captions to behave independently—they are created, their mode defined and rules defined for their update and removal. This means that it is possible to have captions of different modes concurrently within a scene. A *captionManager* is used to keep track of each of the captions in the scene and update them where necessary. This allows the user to override their set mode, and handle basic collision avoidance within the scene. The user is given a choice of how the *captionManager* can remove the captions from the scene. This can be set to the time defined in the caption file, a delay can be added, or it can be specified the maximum number of captions to be displayed. In this case the oldest caption is removed once the maximum threshold is reached.

**Fig. 6:**
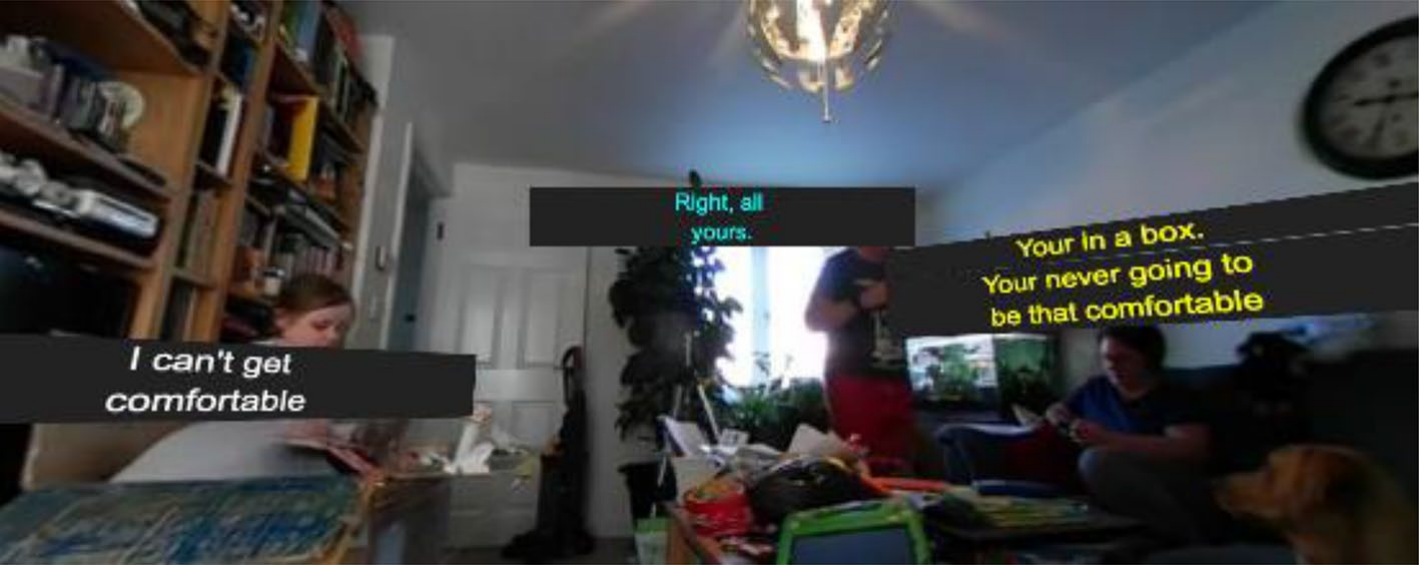
Captions stacked to avoid collisions

**Fig. 7:**
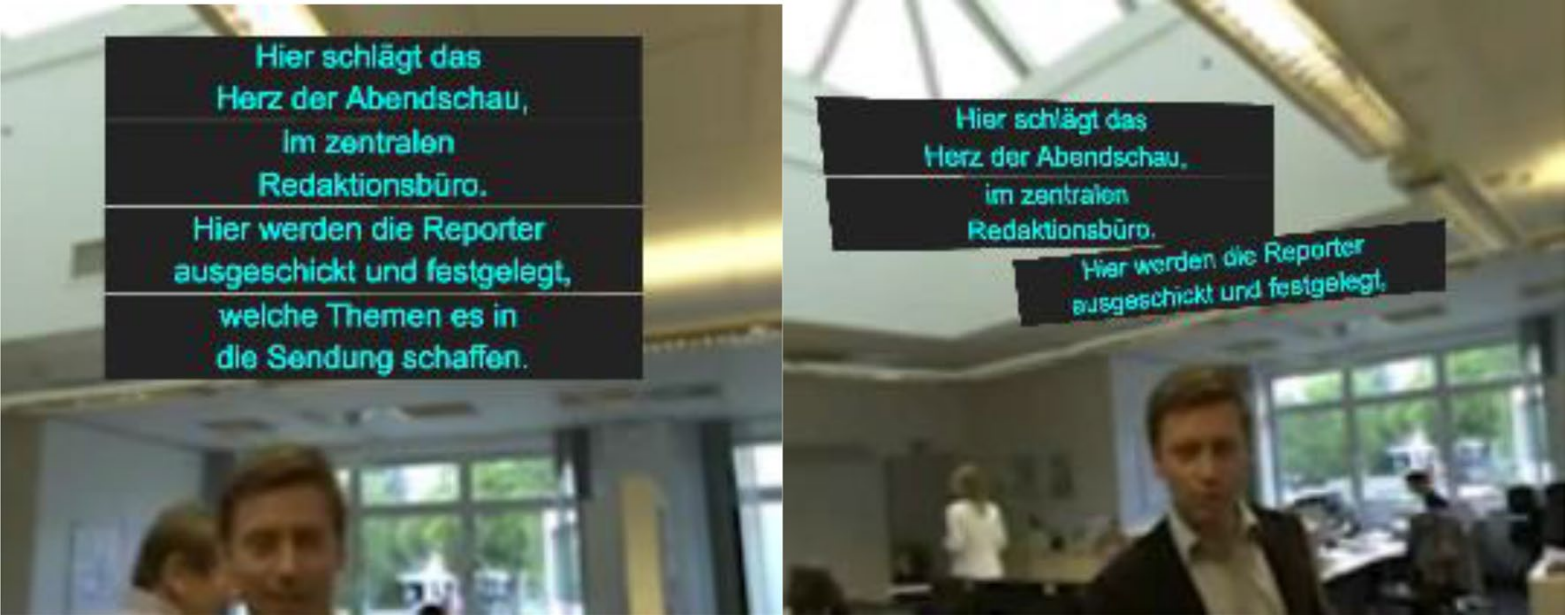
Fixed captions are stacked to avoid collision (left: horizontal position remains where the caption was rendered, right: the stack realigned when it gets updated)

Basic collision detection is used to avoid captions occluding each other. For example, when one character is speaking, but previous captions remain within the scene, if an older caption is not moved, then the new caption is likely to be drawn over the top. Therefore, the *captionManager* implements a *stacking* system as shown in Fig. 6. When a new caption is created, it is added to a *stack*—where a *stack* has been created for each character in the scene, plus an additional stack for *head-locked* captions. When a caption is added to a stack, the *captionManager* iterates through each of the captions in the stack, from newest to oldest and increasing the azimuthal position by the existing height of the stack. This effectively moves the old captions up and places the new captions underneath, replicating the traditional roll-up approach. As only the vertical position is updated, if the character speaking is moving, the horizontal position of captions indicates the path the character has taken. However, there is also an option in the interface to force each stack to realign when it is updated, as shown in Fig. 7.

**Fig. 8:**
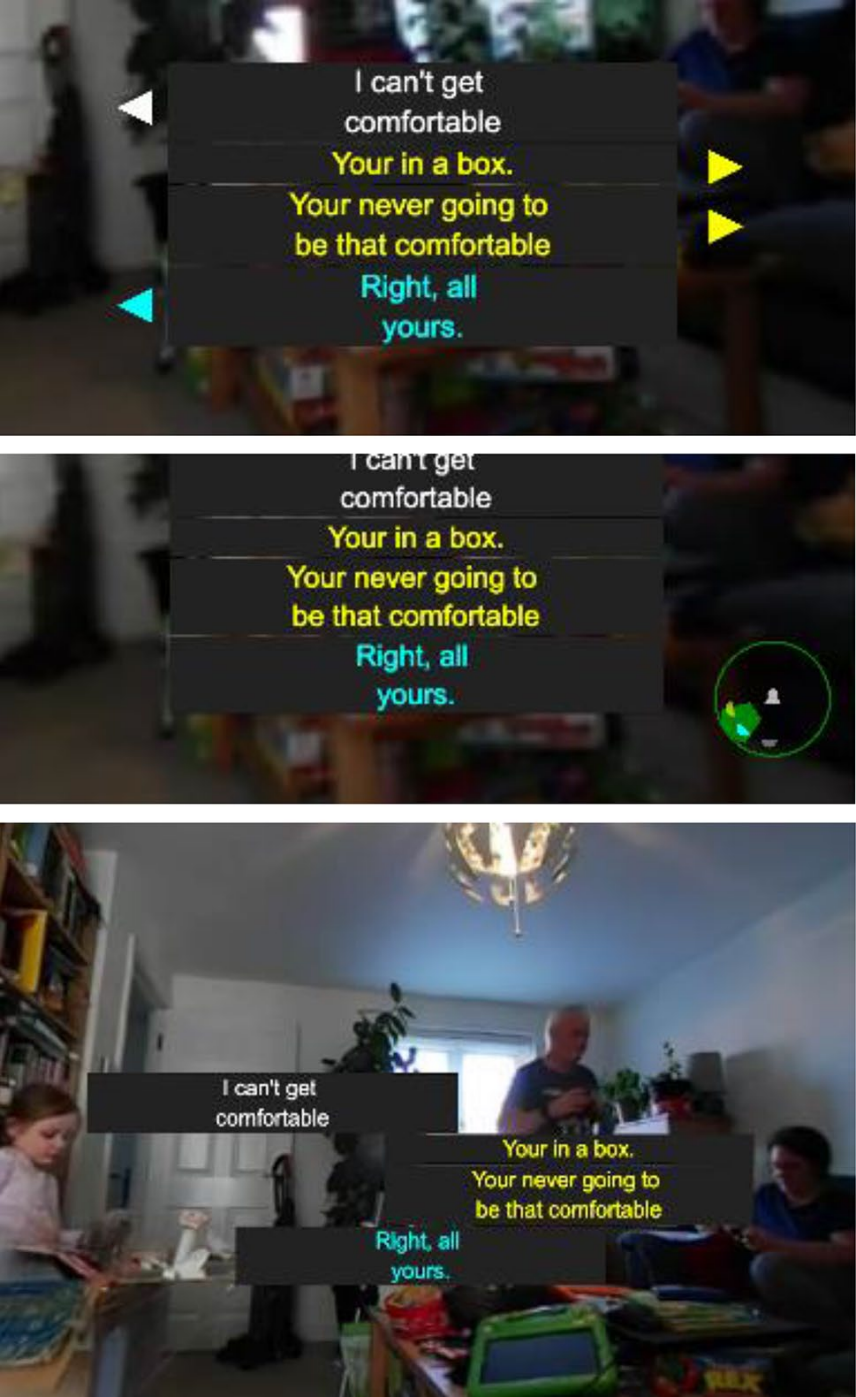
Enhanced guides extend the ImAc approach to support multiple captions (top: arrows, middle: radar and bottom: justified)

Each of the stacks is grouped, allowing the user to apply an offset on each axis, allowing the entire stack to be repositioned. For example, the captions can be moved upwards to place the captions above the person speaking, rather than on top of them. To support the location of multiple captions, the guides were also extended. Each caption object contains its own guide components, so when the ImAc arrow, or ImAc radar mode is enabled in a *head-locked* mode, each caption can display its own guide. As shown in Fig. 8, in the case of the ImAc arrow, each caption can display its own arrow; however, in the case of the radar, an additional overlay is added for each caption target. The opacity of each caption in the radar is reduced as the caption gets older.

### Extended caption viewing time

**Fig. 9:**
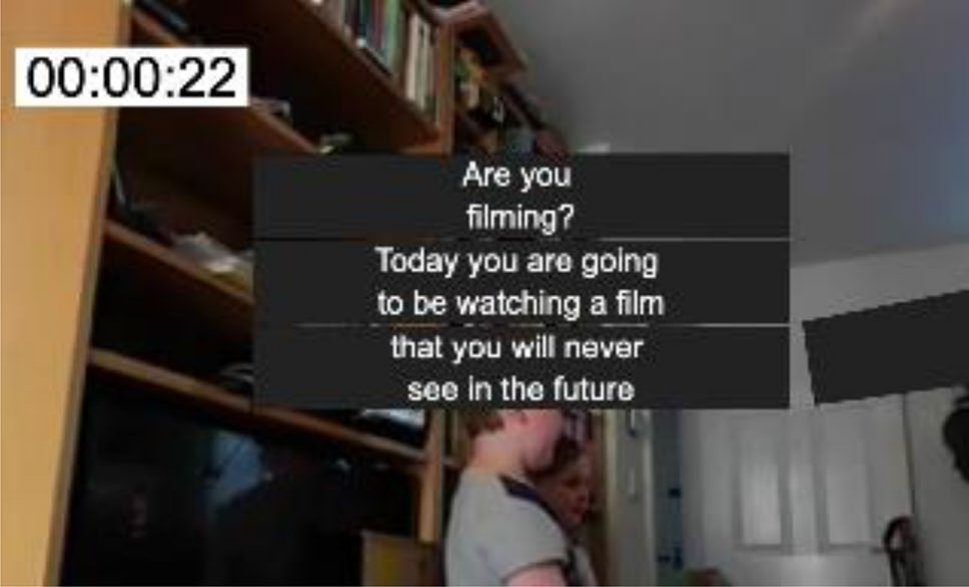
A customizable timecode can be added to the user’s viewpoint

Additional tools were also added, such as a *timecode* display in the view window. This can be fully customized for style and position, in order to help the user understand where they are temporally whilst immersed, as shown in Fig. 9. Also, parameters such as animation speed and offset position are all exposed to the user through the graphical user interface (GUI). An additional option to lock the caption azimuthally was also added to force the captions to remain on the horizon. This may be helpful to those users who find it difficult to look up and down.

### Responsive captions

In previous work, we developed a JavaScript library for managing responsive captions [25]. This library allows for captions to be dynamically restructured into different lengths. This is done by following the principles of text flow and line length, informed by the semantic mark-up along with styles to control the final rendering.

**Fig. 10:**
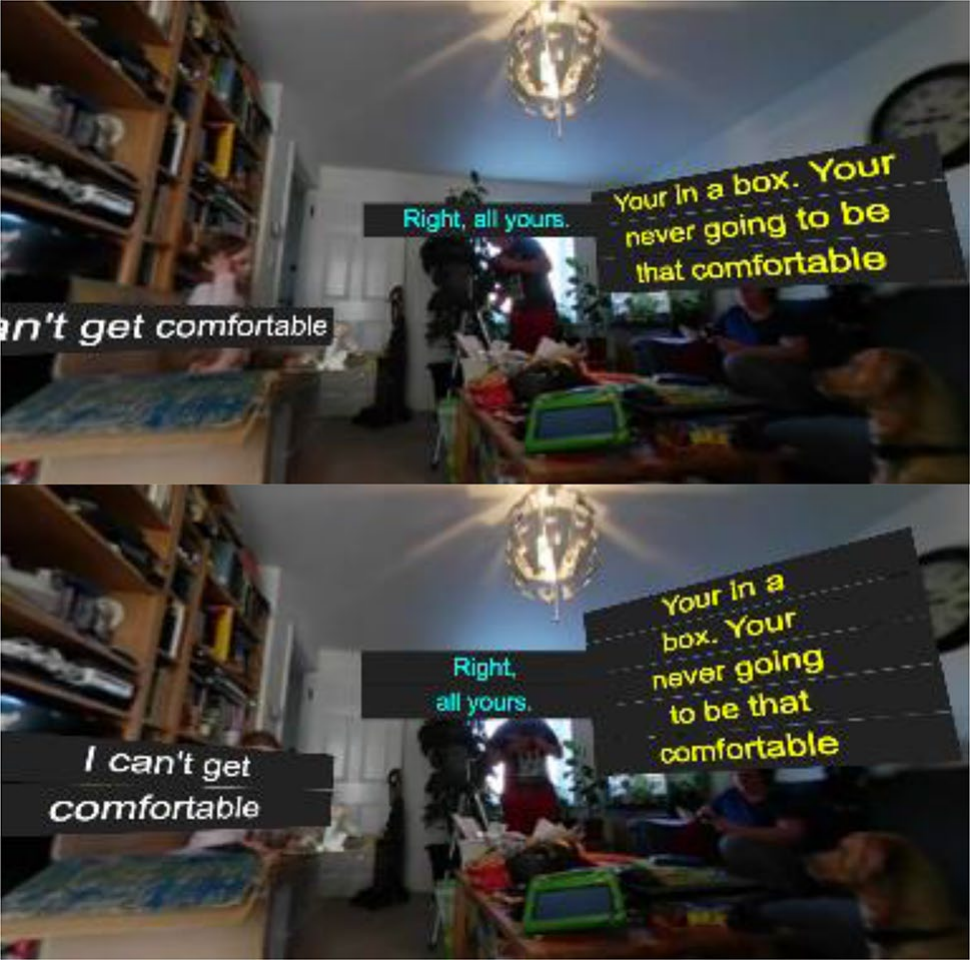
The responsive caption library restructuring the length of the captions to a maximum character length (left: 25 characters, right: 12 characters)

Captions are re-blocked by adhering to the number of characters that can fit into the display container at the chosen font size. Firstly, each paragraph is recombined, based on a unique speaker. A best-fit algorithm then breaks each paragraph up to individual captions in order to fit the container. Due to the nature of the changing font size, this may provide more or less captions than the originally authored; however, as the number of words remains the same, the reading speed never changes. As words are evenly distributed, it also avoids leaving orphaned words, as shown in Fig. 10.

**Fig. 11:**
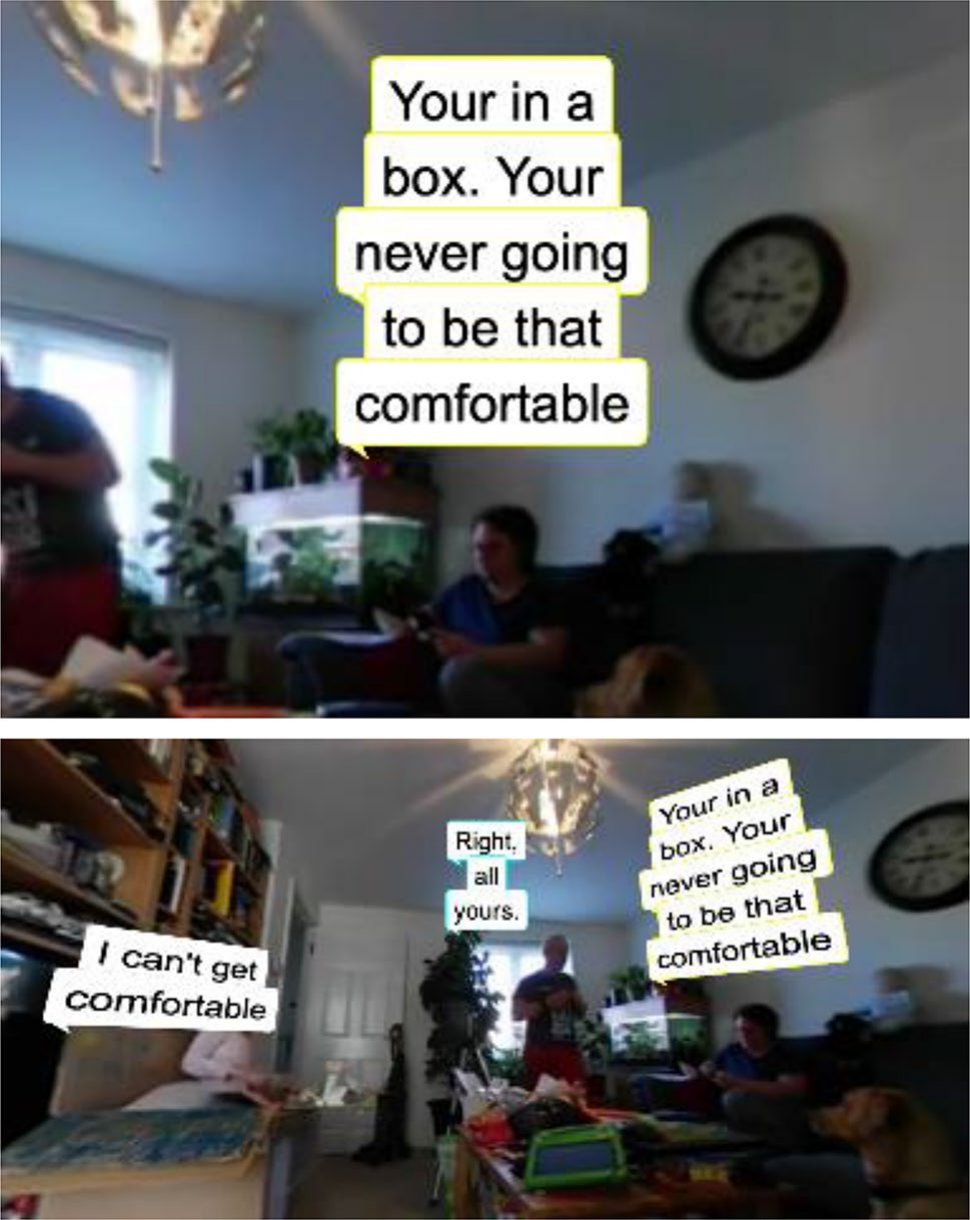
A customizable timecode can be added to the user’s viewpoint

This approach is particularly effective when adapting content from traditional television displays into an immersive environment, such as rendering the caption in a speech bubble attached to a character, or for instance, if one wishes to reduce the width of the caption in order to make room for other captions or graphics, as shown in Fig. 11.

### Enhanced caption file

**Fig. 12:**
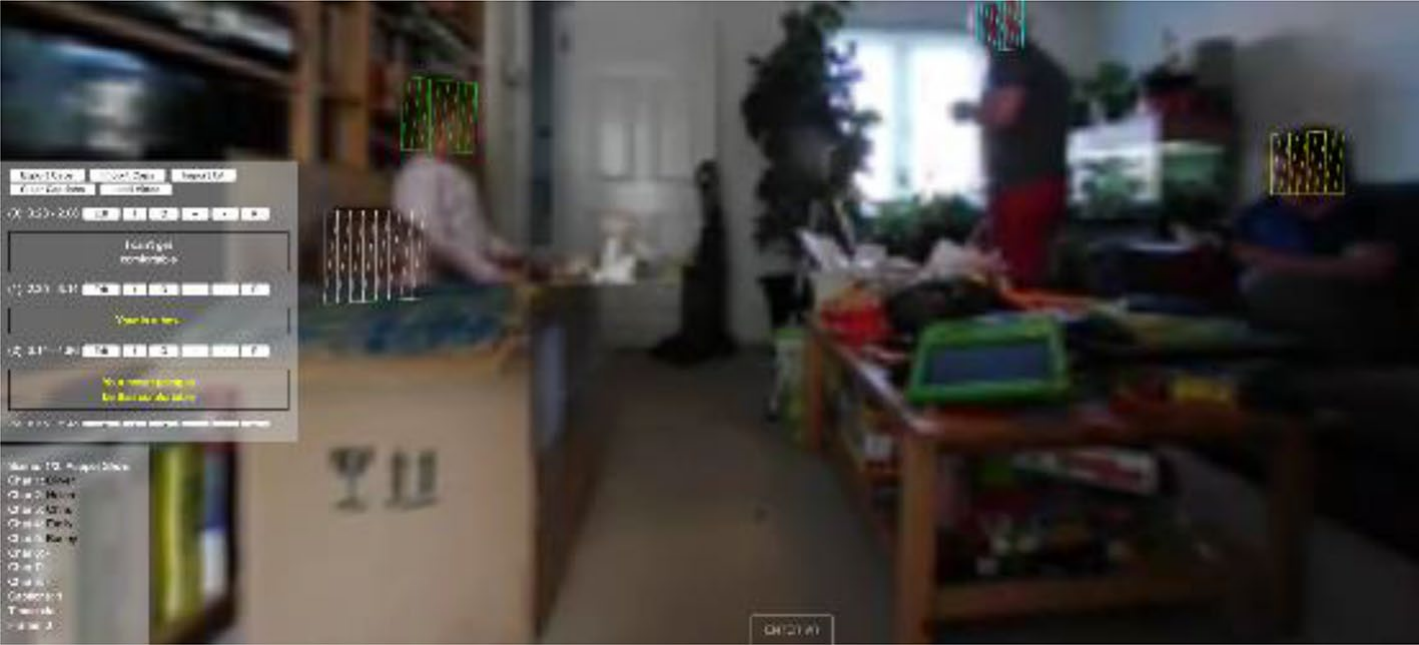
The experimental caption files contain tracked location for each character and the framework provides a basic editor for creating the caption files

In order to facilitate future experiments, a custom caption file format has been implemented using a JavaScript Object Notation (JSON) structure. We have tools for importing and exporting to IMSC TTML and importing from a text-based transcript. Our experimental file contains further information such as tracking information for each character in the scene. This provides a frame-by-frame position for the location of each person, and the target can then be tied to a person or object as they move, rather than just the position they are at when the caption is first created. This allows for both a fixed position for responsive captions as we know a start position for each caption we create, or alternatively for a caption to follow a character through a scene. Where no track information is available for a character, an option is provided to interpolate between one caption target location and the next. This is reasonably successful for when a single character is moving and talking, but breaks when characters change. Therefore, there is also an option to restrict the interpolation to a single character and not include the next characters start location. Currently, the additional track information is created manually, by defining keyframes and interpolating between them. Our framework provides a basic editor for adding the track information using a keyboard interface, shown in Fig. 12. In future work, we will explore how computer vision techniques can be used to automatically identify the position of characters within the scene.

## Results and discussion

Testing technology for usability with end users is a standard prerequisite in the development workflow. When the technology designed is related to accessibility, services is a must. The reason is the context where any accessibility service is developed: the UN Convention of Rights of Persons with Disability (2006) with the motto “nothing about us without us”. This leads to a user-centric approach where end users express their needs and expectations. Experience gained from the 3-year research project (ImAc) [1, 26] showed that: (1) end users need to have real stimuli to comment, (2) testing cycles should be shorter, and to these we add (3) the new COVID-19 reality and the challenge to face-to-face testing.

The proposed framework meets the previous three issues with end users having the stimuli in a real VR simulation. This is a step forward to paper prototyping which was used in ImAc. The reason for paper prototyping in ImAc was the fact that no 360º caption editor existed at the time. It was one of ImAc's objectives to develop one. Hence, the very first user requirements were generated in paper [8, 27], which might have impacted the decisions taken towards testing and further developments [11]. Two reasons led to lengthy testing cycles. The first was the process of generating stimuli with different variables, since it was produced as independent 360º movies, not web based. In ImAc stimuli definition and production meant a democratic choice of content, which was then captioned with the editor, then translated to the languages, and finally tested. The second was the number of end users required for each test [8, 27]. This issue is related to the new world health context. COVID-19 has forced all communication-based industries to consider existing communication technology as alternative to traditional media content production and distribution. Testing end users for IT system development is one of the many activities that needs to be redefined under the new situation. The silver lining is that with a framework as the one presented here online testing is a reality. From the comfort of their home, and following all government health and safety regulations, end users can access stimuli from any device.

## Conclusion

There is no standardised solution to captions in immersive environments (VR). Some preliminary VR captions have been developed and tested. These did not lead to conclusive results, on the contrary they requested further testing. One of the lessons learnt from previous user tests was the cumbersome nature of testing in VR. Testing was challenged due to presentation of captions in terms of time to generate stimuli for testing, and testing itself. The aim for this framework is to be used in conjunction with user tests to identify potential solutions by evaluating each of the the different captioning approaches.

If other caption presentations and modes were to be tested, a new testing platform was required, and that triggered the design of the framework presented in this article. By using JavaScript, this framework allows user-test designers to specify which options are available to the users (if any) and define the parameters for the player as it is loaded. This allows for multiple test scenarios to be defined and presented to the user (or even for parameters to be randomized) and built into a larger test setup.

Though it has not been implemented yet, the next stage is for the framework to become an autonomous web self-sufficient testing platform, where the workflow can be tested for ethics and data protection clearing with raw data included. This framework will be a tool not only for UX but also for learning to caption at universities and hopefully start a new trend where content producers will include media accessibility as one more component in their production workflow.

## Data Availability

A demo of the framework is provided at https://www.chxr.org/demo.
